# A comprehensive review of seroma formation, prevention, and treatment approaches

**DOI:** 10.1016/j.jvscit.2025.101879

**Published:** 2025-06-16

**Authors:** Jasmine M. Peng, Koyal K. Ansingkar, Brittany N. Landavazo, Asad F. Shaikh, Maham Rahimi

**Affiliations:** a College of Medicine, Texas A&M University, Bryan, TX; b School of Engineering Medicine, Texas A&M University, Houston, TX; c DeBakey Heart & Vascular Center, Houston Methodist Hospital, Houston, TX

**Keywords:** Seroma, Vascular surgery, Inguinal surgery, Prevention, Management

## Abstract

Wound complications after common femoral artery exposure remain a significant source of postoperative morbidity, with seroma formation being a prevalent concern. Seromas can contribute to patient discomfort, delayed wound healing, and an increased risk of infection. Effective prevention and management strategies are essential to mitigate these complications and optimize recovery. This review provides an evidence-based analysis of seroma management after groin surgery, including preventive measures, conservative treatments, and surgical interventions. By synthesizing data from clinical studies, systematic reviews, and expert recommendations, this article serves as a practical resource for vascular surgeons and clinicians involved in the postoperative care of patients undergoing vascular procedures.

Groin exploration surgery is performed commonly for conditions such as hernia repair, lymph node dissection, and vascular procedures. In the United States, approximately 800,000 inguinal hernia repairs are conducted annually.^1^ Despite advancements in surgical techniques, seroma formation remains a frequent postoperative complication. Seromas are abnormal collections of serous fluid that accumulate in dead spaces created during surgery, typically consisting of plasma and lymphatic drainage.^2^^,^^3^ Although extensively studied in plastic surgery, where they commonly complicate procedures such as mastectomy with or without axillary lymph node dissection,^3^^,^^4^ seromas remain poorly characterized in vascular surgery. The incidence of seroma formation after mastectomy or axillary lymphadenectomy has been estimated at 24.2%,^5^ although one study reported seromas in up to 22% of patients after femoral artery access.^6^ However, data on seroma incidence in vascular procedures remain limited.

The exact pathogenesis of seroma formation is not understood fully, although both lymphatic leakage and acute inflammation have been implicated.^4^ Early theories attributed seromas primarily to disrupted lymphatic drainage, and studies initially correlated wound drainage volume with seroma formation and lymphedema.^7^ However, emerging evidence suggests a more complex interplay involving inflammatory exudates and tissue shear-induced inflammation.^4^ Patient-specific risk factors, such as age and body mass index, have shown inconsistent associations with seroma development.^3^ These findings indicate that both lymphatic transudation and inflammatory processes contribute to seroma formation.^4^

Various intraoperative and postoperative strategies have been described to prevent and manage seromas, including negative pressure wound therapy, sclerotherapy, lymphangiography, and suction drainage.^8^ Although seromas in plastic surgery are often considered a normal part of recovery, they pose greater concerns in vascular surgery owing to potential complications such as vascular graft infection, mass effect causing pain and discomfort, and the need for surgical intervention increase risk of limb loss.^8^ Given the clinical significance of seromas in vascular procedures, this article aims to provide an evidence-based review of their prevention and management.

## Methods

A literature search was conducted using the keywords “seroma,” “seroma formation,” “seroma formation vascular surgery,” and “seroma prevention.” A total of 48 article were selected based on relevance. Studies published between 1995 and 2024 were included if they addressed the prevention and/or management of seroma. Papers not written in English were excluded. Various types of literature, including case reports, cohort studies, randomized controlled trials, and systematic reviews, were included without restriction based on surgical specialty. Relevant findings were extracted from vascular surgery, general surgery, and plastic surgery. The selected data were categorized into preventative measures and postoperative management and further subdivided by intervention type, including dissection techniques, closure methods, sclerotherapy, lymphangiography, drains, wound care, and secondary management strategies.

## Results

### Preventive measures

#### Lymphatic leaks and preservation techniques

Lymphatic channels are challenging to identify intraoperatively owing to their colorless and minute nature. Techniques such as Evans blue dye application or thigh massage can aid in visualizing these structures. Early detection and timely surgical management of large, actively draining, or suspected infected seromas, through ligation or repair of leaking lymphatic vessels, significantly decrease the risks of prolonged hospitalization and infection, particularly in cases involving prosthetic grafts that often require early intervention.^9^

Lymphatic leaks are a notable complication of arterial reconstruction in the groin, with an incidence ranging from 1.8% to 18.9%.^10^ These leaks increase the risk of vascular graft infections, requiring prompt and effective management. They typically present within 24 hours postoperatively as continuous skin drainage or soft tissue collections. Although some cases resolve spontaneously, predicting which will self-resolve remains challenging. Preventive strategies include meticulous ligation of visible lymphatic vessels during initial groin dissection. However, injury to small lymphatic vessels is often unavoidable, even with optimal surgical techniques.

Emerging technologies, such as the LigaSure electrothermal bipolar sealing device, have shown promise in decreasing lymphatic leak rates. A study by Umeda et al^11^ reported a 0% rate of lymphatic leakage in inguinal or ilioinguinal lymph node dissection procedures for skin cancer when LigaSure was used, compared with conventional techniques. Similarly, Ikeda et al^12^ demonstrated a reduction in seroma formation when LigaSure was applied during axillary lymph node dissection and mastectomy. Leović et al described that after head and neck surgery, large-volume (>500-1500 mL/day) postoperative chyle leaks lasting more than 4 to 5 days are unlikely to resolve without intervention, necessitating further treatment.^13^^,^^14^

The management of lymphatic leaks depends on the severity and patient condition. This approach is often subjective and clinical, with noninfected leaks typically addressed conservatively with rest, antibiotics with skin flora coverage, nutritional support, and local compression with either sterile dressing or, if needed, compression garments. However, discharge from surgical incision or those persisting beyond one week may require surgical reexploration with selective ligation of lymphatic vessels. Although no standardized criteria exist for determining when active intervention is necessary, studies suggest that early operative management can decrease hospital duration of stay and minimize wound infection risk, particularly in cases involving synthetic graft material.^15^

#### Effective hemostasis

Maintaining hemostasis reduces tissue trauma and fluid accumulation. Techniques such as scalpel dissection, hemostatic agents such as fibrin sealants, and suture ligation of bleeding vessels are critical in reducing postoperative seroma formation. Considering recent evidence of a more inflammatory cause for seroma formation, prevention may be achieved by minimizing tissue harm and decreasing the potential for inflammation.

#### Use of drains

Closed suction drains are often used to remove excess fluid from surgical sites and eliminate dead space. However, the effectiveness of drains in preventing seromas is debated, with some studies finding no significant difference in seroma rates between the drained and undrained groups.^4^ A systematic review by Janis et al^4^ showed that, of 14 randomized controlled trials, 4 studies found that closed suction drain use decreased seroma formation, whereas 10 studies found that there was no significant difference. Long-term drain placement can also increase the risk of sinus tract formation, leading to a failed or occluded vascular graft and further increasing the risk of infection.

#### Activity modification

Patients are advised to limit physical exertion and avoid activities that increase intraabdominal pressure to prevent seroma exacerbation or recurrence. One study by Shamley et al^16^ demonstrated that delaying strenuous activities, such as heavy lifting and repetitive arm movements, for 10 to 14 days after breast cancer surgery significantly decreased seroma formation. However, this recommendation often contrasts with the encouragement of early movement after surgery to prevent shoulder stiffness.^16^ It is important to note that, after groin surgery, early ambulation may be less feasible compared with axillary dissections in plastic surgery, which may, in turn, increase the risk of complications such as deep vein thrombosis. Identifying the balance between the need for postoperative movement and the importance of reducing the risk of seroma formation or recurrence requires further research. It should also be noted that this information relates specifically to mastectomy. There are no data regarding activity modifications and their potential to decrease seroma formation in inguinal surgery.

#### Dissection techniques

Several dissection techniques have been investigated for their role in reducing seroma formation. In a randomized controlled trial, Gong et al^17^ demonstrated that sharp dissection with lymph vessel ligation significantly decreased the rate of seroma formation in mastectomy patients, reducing the incidence from 14% to 2%. Additionally, patients who underwent sharp dissection with lymph vessel ligation exhibited lower daily drain output, suggesting that this technique may be an effective strategy for minimizing seroma formation.

LigaSure, an electrothermal bipolar sealing device, has shown promise in sealing both blood vessels and lymphatic channels. The LigaSure device creates a permanent seal by delivering a controlled amount of bipolar energy to vessel walls. It operates through an automated cycle lasting 2 to 4 seconds and is capable of sealing vessels up to 7 mm in diameter without the need for clips or ties. This technology is particularly advantageous for minimizing thermal spread to surrounding structures while ensuring reliable hemostasis of the targeted vessel. Umeda et al^11^ reported that using LigaSure during inguinal lymph node dissection significantly reduced the incidence of postoperative lymphatic leaks from 37% to 0%. Furthermore, postoperative lymphatic drainage volume was also reduced, supporting the effectiveness of LigaSure in preventing seroma formation.

Conversely, some studies have raised concerns regarding the use of electrocautery during dissection. Porter et al^18^ compared scalpel dissection with electrocautery in mastectomy patients and found that, although electrocautery reduced intraoperative blood loss, it was associated with a significantly higher incidence of seroma formation. Additionally, drain type influenced seroma rates, with Jackson-Pratt drains correlating with a higher incidence of seroma formation compared with Blake drains.^18^

Valença-Filipe et al^19^ further examined scalpel dissection vs monopolar diathermocoagulation in coagulation mode in patients undergoing abdominoplasty. Their findings indicated that scalpel dissection resulted in lower daily drain output and reduced seroma formation compared with monopolar diathermocoagulation.^19^

Overall, the literature suggests that scalpel dissection is superior to blunt techniques or electrocautery in preventing seroma formation. Patients who underwent sharp dissection techniques experienced fewer postoperative complications and lower daily drain output. LigaSure showed the most promise in decreasing both seroma formation and lymphatic drainage postoperatively. Implementing these techniques in groin procedures and femoral artery access surgeries may help reduce seroma formation rates.

#### Incisional negative pressure wound therapy

Incisional negative pressure wound therapy (iNPWT) has gained popularity for decreasing postoperative wound infections and dehiscence. iNPWT is applied over closed incisions and through the use of negative pressure it reduces superficial tension, evacuating underlying fluid and enhancing capillary perfusion, ultimately promoting wound healing.^20^ One study by Rezk et al^21^ outlined a decrease in lower extremity bypass surgical wound dehiscence after using iNPWT, although no significant decrease in surgical site infection, defined by ASEPSIS score (Additional treatment, Serous discharge, Erythema, Purulent exudate, Separation of deep tissues, Isolation of bacteria, and Stay), was noted. However, Benrashid et al^22^ showed that the use of closed surface NPWT devices over lower extremity or infrainguinal incisions showed a significant decrease in surgical site infections compared with the conventional dressings.

Recent studies have investigated technical modifications to improve its efficacy. For instance, Mehdorn et al^23^ tested variations of the PREVENA system in an ex vivo model. They found that incorporating wound gauze or suction drain tubes improved seroma evacuation, achieving near-complete fluid removal compared with only 56% evacuation with standard iNPWT.^23^ Suction tubes were particularly effective for deeper seromas, decreasing the risk of deep wound infections.

Additionally, a case series by Abai et al^24^ described three patients readmitted for lymphatic leaks and lymphoceles after vascular interventions. All were treated successfully with NPWT, with fluid leakage resolving within 14 days on average. A separate analysis of outcomes after vacuum-assisted closure therapy for groin infections revealed high rates of vascular graft preservation, underscoring the benefits of this approach. By addressing lymphatic leaks, reducing empty space, and accelerating wound healing, iNPWT may play a critical role in managing postoperative complications and improving surgical outcomes.

#### Porcine dermal collagen: Decreased inflammation

Porcine dermal collagen, derived from the acellular pig matrix, closely resembles human dermis in structure. Marketed under the brand name Permacol, it is an acellular matrix mesh composed of cross-linked collagen fibers treated with hexamethylene diisocyanate to enhance stability and decrease collagenase degradation.^25^ Typically, the matrix is sutured in place to cover the wound, promoting fibroblast infiltration, growth, and neovascularization. It has been used widely in repairing abdominal wall defects, burn treatment, and various reconstructive procedures for the pelvis, head, and face.^26^

In a study investigating its role in decreasing seroma formation after mastectomy in Wistar rats, researchers found an inverse correlation between the amount of porcine dermal collagen applied and postmastectomy seroma volume.^27^ The experiment divided rats into three groups: no collagen application, collagen applied to 50% of the mastectomy area, and collagen applied to 100%.^27^ After 10 days, significant differences in seroma volumes were observed among the groups. A literature review accompanying this study suggested that materials decreasing dead space and enhancing fibrosis outperform sclerosing agents in minimizing postoperative seromas.

#### Fibrin sealant tissue adhesive

Fibrin sealant, or fibrin glue, is a plasma-derived hemostatic agent combining thrombin and fibrinogen. It mimics the final stage of the coagulation cascade, interacting with ionized calcium to form an insoluble clot. Fibrin sealants are used commonly to prevent seroma and lymphocele formation, particularly after lymphadenectomy.

A randomized controlled trial demonstrated a significant decrease in both the incidence and volume of seroma formation after inguinal lymph node dissection when fibrin sealant was applied.^28^ However, a meta-analysis of six randomized controlled trials comparing fibrin sealant with standard closure techniques after groin dissection found no statistically significant differences in seroma incidence.^29^

#### Talc sclerodesis

Subcutaneous talc is used frequently to prevent seroma formation, particularly after axillary lymph node dissections. Talc is a naturally occurring hydrous magnesium silicate containing magnesium, silicon, oxygen, and hydrogen, with varying trace elements such as calcium and aluminum.^30^ It is recognized widely for its use in chemical pleurodesis to prevent recurrent pleural effusion or pneumothorax. Talc sclerotherapy can be performed either by applying the dry powder directly to the operative site or by injecting a mixture of talc and local anesthetic prior to closure, with the use of a drain left to the surgeon's discretion.

Although some studies attribute talc's efficacy to its ability to induce significant inflammatory responses that promote cytokine and adhesion molecule production (eg, interleukin-8, vascular endothelial growth factor, and transforming growth factor-beta), others argue that talc works by roughening tissue surfaces, decreasing shear without causing substantial inflammation.^4^ Other sclerotherapy agents, such as povidone iodine, alcohol, and doxycycline, have been effective in managing postoperative lymphoceles across various surgical contexts, including transplant, vascular, and breast surgeries.^31^

The application of talc in vascular surgery is less well-documented. However, Metcalfe et al^32^ reported a case in which sterile talc was used intraoperatively to manage a high-output groin seroma after an endovascular abdominal aortic aneurysm repair. Application of sterile talc decreased serous output from the wound, allowing for the discontinuation of negative pressure dressings within 4 days.^32^

These techniques—porcine dermal collagen, fibrin sealant, and talc sclerodesis—offer promising approaches to managing seromas and lymphatic leaks, with varying mechanisms of action and effectiveness based on clinical context. It is important to recognize that the use of fibrin sealants and talc sclerodesis may promote scar tissue formation, which can complicate future reoperations. This potential drawback should be considered carefully when selecting a treatment strategy for patients who may require multiple groin interventions, particularly in vascular surgery cases. The Table highlights these methods with the percent reduction in seroma formation.

**Table:** Reduction in seroma formation using various techniques as outlined in the literature

Technique	Reduction in seroma formation	Type of study	Sample size (total; treatment)
Electrothermal bipolar cautery (LigaSure)	100%	Retrospective	n = 58; 10
Doxycycline sclerodesis (seroma management)	87.94%	Retrospective	n = 38
		Case report	n = 1 case report
Porcine dermal collagen (Permacol)	46.17%-86.88%	RCT animal	n = 18; 6
Quilting sutures	13.0%-56.0%	RCT human	n = 108; 54
		Prospective	n = 27; 11
		Observational	n = 82; 41
Talc sclerodesis	18.1%-33.0%	Clinical trial	n = 180; 74
		Comparative animal	n = 6
		Case report	n = 2 case reports
iNWPT	6.3%-44.4%	Ex vivo experimental	n = 1
		Retrospective	n = 151; 73
		Case report	n = 2 case reports
Fibrin tissue sealant adhesives	0.17%-37.5%	RCT human	n = 32; 16
		Systematic review	n = 6 RCT studies
Lymphatic ligation	12.00%	RCT human	n = 201; 101

*iNWPT,* Incisional negative wound pressure therapy; *RCT,* randomized controlled trial.

#### Other closure techniques

Muscle flaps can be used prophylactically to decrease the rate of seroma formation. Fischer et al^33^ describe muscle flap use specifically in the case of femoral artery access through a groin incision. The patients who received these prophylactic muscle flaps had fewer postoperative complications, including a 0% seroma formation rate compared with a 7.2% seroma formation rate in patients with open vascular procedures without prophylactic muscle flaps.^33^ This technique also involved fewer wound complications.

Quilting sutures, a technique that can be used to decrease dead space, have also been shown to reduce the formation of seroma by Daltrey et al^34^ in patients undergoing latissimus dorsi reconstruction of the breast. Titley et al^35^ described that quilting sutures can also be used with latissimus dorsi flaps in the lower extremity. After using quilting sutures in a variety of procedures, including both breast and the lower extremity, they identified that the incidence of seroma was decreased significantly.^35^

Although the techniques described can decrease seroma formation significantly, some cases remain unavoidable. In these instances, various management strategies are necessary, ranging from conservative approaches to surgical interventions. The following sections outline evidence-based methods for addressing seromas when they occur.

### Infected vs noninfected seromas

Effective management of seromas requires distinguishing between noninfected and infected presentations. Noninfected seromas typically appear as a soft, painless lump near the surgical incision site, without associated drainage, erythema, or significant discomfort. In contrast, infected seromas are often painful and may present with erythema and drainage. In the groin, infections are classified commonly using grading systems such as the Szilagyi and Samson scales.^36^^,^^37^

The Szilagyi grading scale categorizes infections by the depth of tissue and arterial involvement^36^:

• Grade I involves the dermis only.
• Grade II extends into subcutaneous tissue.
• Grade III affects the graft itself.

The Samson grading scale expands on this, detailing five groups of infections^37^:

• Group 1: Limited to the dermis.
• Group 2: Extends into subcutaneous tissue.
• Group 3: Involves the arterial graft body but spares the anastomosis.
• Group 4: Surrounds the anastomosis without bacteremia or bleeding.
• Group 5: Fully involves the graft and anastomosis, often accompanied by septicemia.

The treatment of infected seromas requires surgical intervention. For cases with external drainage, surgical exploration and debridement are performed until a clean wound bed is achieved. If the infection is superficial and does not involve the arterial graft, wound vacuum-assisted closure for a few days of wet to dry povidone-soaked dressing or muscle flap reconstruction may suffice. However, if the infection extends to the arterial graft, revascularization is necessary. Options include in situ reconstruction with rifampin-soaked Dacron, cryopreserved conduit, autologous vein, or extra-anatomical bypass, often through the obturator foramen. The choice of method depends on the infection's virulence and severity.

For intact skin without overt signs of infection, diagnosis becomes more challenging. Differentiating infected seromas from cellulitis adds complexity; it is not always clear if surgical intervention is warranted. Careful clinical judgment and additional diagnostic tools are essential in these cases to identify the involvement of the vascular graft and the extent of the fluid collection.

### Nonoperative management

Small seromas (<75 mL aspirated fluid) with minimal symptoms typically resolve spontaneously with conservative measures such as bed rest, elevation, compression, and prophylactic antibiotics. Studies report success rates of 75% to 90%, with resolution occurring in 14 to 24 days.^38^ However, retrospective data suggest that early lymphatic ligation shortens resolution time and decreases recurrence compared with conservative therapy.^39^ The following sections discuss the nonoperative management of seroma.

#### Compression dressings

Applying compression garments or binders helps to reduce fluid accumulation by increasing tissue pressure and promoting lymphatic drainage, facilitating seroma reabsorption. There is limited literature on the use of compression dressings specifically for femoral seromas; however, existing studies suggest that they may offer beneficial outcomes specifically in plastic surgery. In one study by Fabro et al,^40^ compressive taping was found to aid reabsorption of seromas secondary to breast cancer surgery significantly, demonstrated by a decrease in aspiration volume before and after compression taping for 5 consecutive days. The authors concluded that compressive taping is a safe and noninvasive treatment of postoperative seroma with high adherence and patient satisfaction.^40^ However, some other studies have shown that compression dressings after axillary lymphadenectomy are ineffective in reducing seroma formation when compared with controls, which may be due to a lack of standardized compression dressing application procedure and variation between patient cases.^41^

#### Serial aspiration

Larger seromas (>75-100 mL) often cause pain, or functional impairment, necessitating aspiration. Percutaneous aspiration under ultrasound guidance is effective for draining fluid in asymptomatic patients. However, repeated aspirations may be needed, posing risks of introducing bacteria into the seroma cavity or surrounding tissue injury. Patients requiring serial aspirations for more than 40 days after their initial operation have a higher risk for surgical revision, with one study noting that those with aspirations after more than 40 days had a nine times higher risk of requiring surgical repair than those with aspirations done before 40 days.^42^ Chronic seromas can form encapsulated pseudocysts requiring open drainage and debridement, although such cases are rare and appear only in case reports.^2^ Thus, the postoperative treatment of seromas should be performed in an efficient manner to decrease reoperations and infections.

A safe, outpatient seroma drainage method using an angiocatheter has been described.^43^ A 14G catheter was inserted into the seroma cavity and connected to suction tubing for continuous low-pressure evacuation. This technique minimizes risks, such as puncturing implants during breast reconstruction surgeries; however, this technique may not be optimal in the femoral region, specifically when using a prosthetic graft.

These approaches, ranging from conservative management to advanced surgical interventions, provide a comprehensive framework for effectively managing chronic seromas and lymphatic leaks.

### Surgical interventions

For seromas that are large, symptomatic, recurrent, or unresponsive to conservative management, surgical options may be necessary.

#### Seroma evacuation

Surgical drainage or evacuation of persistent seromas can be performed under local or general anesthesia. This approach may involve reopening the previous incision or creating new incisions to access the seroma cavity after ultrasound evaluation. These procedures often involve looking for a source of the lymphatic leakage, ligating the relevant lymphatic channels, and potentially placing additional clips and cauterizing other areas of concern within the seroma cavity.^33^ Drains can also be placed to prevent further fluid buildup.

#### Lymphangiography

Lymphangiography has been a tool for mapping lymphatic anatomy since the 1930s. The technique gained prominence in 1955 with Kinmonth et al's^44^ description of the Kinmonth method, which involved cannulating a lymphatic channel in the foot and injecting blue dye. Historically, it was used for lymphoma staging, distinguishing inflammatory from neoplastic processes, and assessing metastasis and chemotherapy responses. However, its invasiveness, technical difficulty, and time requirements have led to the adoption of ultrasound-guided lymphangiography.^45^^,^^46^

One study described using SPY lymphangiography to manage a persistent high-output lymphatic leak.^47^ After failed management with a sartorius muscle flap, indocyanine green dye was injected into the first toe web space and anterior ankle to identify large lymphatic channel leaks. These channels were ligated successfully using LigaSure and suture ligation, with SPY imaging confirming complete resolution. Postoperatively, a 15F drain was placed and removed on day 7, and a wound vac was maintained for 2 months. Lymphatic ligation has been shown to be one of the most effective interventions for lymphatic leaks, with success rates ranging from 75% to 100% and resolution times averaging 0 to 9 days.^38^

#### Suture techniques

A case series highlighted two instances of rapidly reaccumulating seromas resolved with external quilting sutures using 1/0 silk.^48^ This method, often used in plastic surgery for procedures such as mastectomy and latissimus dorsi flap harvests, was adapted for groin dissections. Patients with rapidly reaccumulating groin seromas were taken back to the operating room for the placement of sterile sponges with quilting sutures with a corrugated drain and fibrin sealant.^48^ Quilting sutures tied over sterile sponges were removed once drainage was less than 10 mL within 24 hours. This technique showed promising results in the management of persistent groin seromas; however, it is important to note that it would require returning to the operating room, and this approach should be considered for high-risk patients with multiple comorbidities commonly treated in vascular surgery.

#### Sclerotherapy

Aspiration of the seroma followed by injection of a sclerosing agent promotes fibrosis and closure of lymphatic channels. A systematic review by Sood et al^49^ showed that talc, tetracycline antibiotics, erythromycin, fibrin glue, ethanol, polidocanol, OK-432 (Picibanil), and povidone-iodine can all be used as sclerosants for the chronic management of seroma. All of these agents showed success and can be used multiple times in the case of recurrence.^49^ Although sclerotherapy is effective for refractory seromas, risks include skin injury and infection, particularly with ethanol and talc use. The presence of an infected seroma before sclerotherapy may decrease its effectiveness.^49^ This management option may be suitable for patients with recurrent seromas that have not responded to aspiration and continue to reaccumulate.

#### Chronic seroma: Doxycycline sclerotherapy and NPWT

A case report described a patient with recurrent chronic seromas after undergoing multiple abdominal surgeries, including an appendectomy, incisional hernia repairs, and panniculectomy.^50^ The seroma was successfully managed using doxycycline sclerotherapy combined with NPWT via the KCL-V.A.C. Ultrasystem. The procedure involved placing white foam within the open wound, extending approximately 3 cm into the seroma cavity, covered with GranuFoam and a Veraflo dressing.

A low-volume, high-frequency sclerosant infusion technique was used by instilling 60 mL of doxycycline solution (20 mg/mL) into the wound every 2 hours using the NPWT device. After instillation, the patient was encouraged to ambulate to enhance solution distribution. The device then switched to negative pressure mode to aspirate the remaining sclerosant, repeating the cycle six times. Continuous negative pressure was maintained afterward to promote cavity collapse and adherence of wound surfaces. An abdominal binder was applied to further reduce seroma cavity size. The seroma resolved within 20 days, after which a Prevena wound vac was applied.^50^

However, a systematic review by Janis et al^4^ published in *Plastic and Reconstructive Surgery* noted that prophylactic use of sclerosing agents like doxycycline could increase seroma formation paradoxically, owing to the inflammation induced by these agents, which roughens the seroma capsule and facilitates cavity collapse. This finding aligns with findings that tissue inflammation and shearing are major contributors to seroma development.

#### Muscle flaps

In cases of recurrent seromas or poor tissue integrity, a muscle flap using adjacent tissues can help to close the seroma cavity and prevent fluid reaccumulation. Historically, sartorius muscle flaps have been used in groin procedures because of their proximity to the relevant vessels. Recently, rectus femoris muscle flaps have been proposed as an alternative muscle flap; they have sufficient blood supply and have been used successfully in the management of wound complications after vascular groin procedures.^51^ Additionally, gracilis flaps and rectus abdominis flaps have been used to manage groin surgical wound infections.^33^^,^^38^ Last, it should be noted that muscle flaps can be used prophylactically to decrease the likelihood of seroma formation, as well as for the surgical management of seromas after inguinal surgery.^52^ A case report by Scaglioni et al^53^ also showed success with a pedicled superficial circumflex iliac artery perforator flap, which is a lymphovenous anastomosis that allowed for restoration of lymphatic drainage pathways.

## Conclusions

Effective prevention of seroma formation is crucial for improving postoperative outcomes. Lymphatic ligation is a first-line surgical approach for preventing seromas and has shown promise as a safer alternative to electrocautery. The most effective strategy for the prevention of seroma as shown by the literature was the use of LigaSure, an electrothermal bipolar cautery device, during dissection in the initial inguinal surgery. Quilting and progressive tension sutures significantly decrease seroma formation by minimizing dead space. Sclerosing agents such as fibrin sealant, talc, and doxycycline have been evaluated for seroma prevention. Fibrin sealant, which mimics coagulation to form clots, has shown mixed results. Talc and doxycycline act by inducing inflammation or altering tissue surfaces, although their effectiveness remains unclear in vascular surgery. Lymphangiography has evolved from invasive techniques to minimally invasive, ultrasound-guided approaches. Lymphangiography has also demonstrated success in resolving persistent lymphatic leaks when combined with advanced wound care. iNWPT has also gained popularity for reducing wound infections and dehiscence.

Small seromas often resolve spontaneously and can be managed conservatively with bedrest, compression, and prophylactic antibiotics. Nonoperative management boasts success rates of 75% to 90% with resolution typically within 14 to 24 days. However, early surgical intervention, such as lymphatic ligation, is favored for large-volume or high-output seromas to reduce hospital stays and recurrence. Chronic seromas, which can evolve into encapsulated pseudocysts, may require open surgical drainage and debridement.

Outpatient ultrasound-guided drainage provides effective alternatives for persistent seromas, particularly after breast reconstruction. Evidence also suggests that delaying aspiration beyond 40 days increases the risk of surgical revision. However, there is a risk of inadvertent introduction of bacteria into the seroma cavity, which can lead to infection and further complications.

Seroma formation remains a challenging complication, particularly in vascular surgery, with the risk of vascular graft infection. Evidence-based strategies such as electrothermal bipolar cautery, lymphatic ligation, quilting sutures, and tailored wound care protocols can help to mitigate risks. Future research should aim to refine these methods and develop consensus guidelines for the prevention and management of seroma in vascular surgery.

## Funding

None.

## Disclosures

None.
